# Supplementary material for: Content-rich biological network constructed by mining PubMed abstracts
Source: BMC Bioinformatics. 2004 Oct 8;5:147. doi: 10.1186/1471-2105-5-147 (PMC528731; doi:10.1186/1471-2105-5-147)
Supplement: Additional File 2 — The original results of the above study (non-essential files are deleted to keep the file size under the limit set by BMC bioinformatics). [file 1471-2105-5-147-S2.bz2 › chilibotAdditionalFile2/dip10/574ID7703225E10941/html/NTF3_BDNF.html]

 


 **NTF3** and **BDNF** 
  
Found 1139 abstracts in PubMed, retrieved 10.  
 

 What does Google say? 
 PDF only 
| .edu only 

---

**Interactive relationship** (e.g. stimulation, inhibition, etc)

**Non-interactive relationship** (e.g. studied together, co-existance, homology, etc.)

- Cultured Xenopus spinal cord neurons were treated with a mixture of  **BDNF** , glia derived neurotrophic factor, NT 3  [ **NTF3** ] , and NT 4, in addition to forskolin and IBMX or the cell permeant form of cAMP, to elevate the cAMP level.  Ref: 12832528 J Neurosci, 2003
- Prior exposure to MeHg had no effect on NT 3  [ **NTF3** ]  or  **BDNF**  stimulated PI hydrolysis in the cerebellum.  Ref: 12835095 Neurotox Res, 2000
- Cultured cortical progenitors express  **BDNF**  and NT 3  [ **NTF3** ] , as well as their receptors TrkB tyrosine kinase receptor B and TrkC.  Ref: 12832539 J Neurosci, 2003
- Here, we investigated the role of two endogenously produced growth factors, the neurotrophins brain derived neurotrophic factor  [ **BDNF** ]  and neurotrophin 3  [ **NTF3** ]  NT 3  [ **NTF3** ] , in the early progenitor to neuron transition.  Ref: 12832539 J Neurosci, 2003
- LR or DR alter the expression of the mRNAs for NGF,  **BDNF**  and NT3  [ **NTF3** ]  and CNTF within the visual system.  Ref: 12855194 Brain Res Dev Brain Res, 2003
- NGF,  **BDNF** , NT3  [ **NTF3** ] , and NT4 were localized in the lacrimal gland acini with differing cellular distributions.  Ref: 12882781 Invest Ophthalmol Vis Sci, 2003
- Moreover, trophic effects of OLG conditioned medium were partially blocked by K252a, a trk tyrosine kinase inhibitor, and by neutralizing anti  **BDNF**  or anti NT 3  [ **NTF3** ]  antisera, indicating that neurotrophins may mediate these effects, perhaps in concert with other signals.  Ref: 12843289 J Neurosci, 2003
- We first examined the relative potency of the neurotrophins, brain derived neurotrophic factor  [ **BDNF** ]   **BDNF** , neurotrophin 3  [ **NTF3** ]  and nerve growth factor to prevent glutamate mediated apoptosis.  Ref: 12829403 Neurotox Res, 2002
- Neurons secreted the brain derived neurotrophic factor  [ **BDNF** ]  and neurotrophin 3  [ **NTF3** ]  to induce neuron differentiation, while astrocytes secreted ciliary neurotrophic factor for astrocyte differentiation.  Ref: 12837625 Mol Cell Neurosci, 2003
- In the retina, superior colliculus SC, primary visual cortex V1, hippocampus HIPP and cerebellum CBL, using a ribonuclease protection assay RPA, we examined expression of the mRNAs for nerve growth factor NGF,  **BDNF** , NT3  [ **NTF3** ] , NT4, ciliary neurotrophic factor CNTF and glial cell line derived neurotrophic factor GDNF.  Ref: 12855194 Brain Res Dev Brain Res, 2003
- Western blot analysis confirmed these results, showing that the lacrimal gland expresses NGF,  **BDNF** , NT3  [ **NTF3** ] , and NT4 as well as the NTrs TrkA, TrkB, and TrkC and the p75 protein.  Ref: 12882781 Invest Ophthalmol Vis Sci, 2003
- To determine which of the neurotrophins NTs nerve growth factor NGF, brain derived neurotrophic factor  [ **BDNF** ]   **BDNF** , neurotrophin 3  [ **NTF3** ]  NT3  [ **NTF3** ] , and neurotrophin 4 5 NT4 and their receptors NTrs, TrkA, TrkB, TrkC, and p75, are present in the adult rat lacrimal gland.  Ref: 12882781 Invest Ophthalmol Vis Sci, 2003
- RT PCR showed positive bands at the appropriate sizes for NGF,  **BDNF** , NT3  [ **NTF3** ] , and NT4, and for the receptors TrkA, TrkB, TrkC, and p75.  Ref: 12882781 Invest Ophthalmol Vis Sci, 2003
- Acute exposure to 10 micro M MeHg increased basal PI hydrolysis in cortical slices and increased NT 3  [ **NTF3** ]  and  **BDNF**  stimulated PI hydrolysis in slices from the cerebellum.  Ref: 12835095 Neurotox Res, 2000
- The expression pattern of neurotrophins changes with age, with falling NGF, NT3  [ **NTF3** ]  and NT4 but increasing granule cell  **BDNF** .  Ref: 12879965 Cerebellum, 2002
- We recently found that human amniotic epithelial HAE cells secrete biologically active neurotrophins such as brain derived neurotrophic factor  [ **BDNF** ]  and neurotrophin 3  [ **NTF3** ] , both of which exhibit trophic activities on dopamine DA neurons.  Ref: 12865158 Brain Res, 2003
- Neurotrophin 3  [ **NTF3** ]  NT 3  [ **NTF3** ]  and brain derived neurotrophic factor  [ **BDNF** ]   **BDNF**  stimulated PI hydrolysis in neocortical and cerebellar slices from neonatal rats in a concentration dependent manner 30 1000 ng ml.  Ref: 12835095 Neurotox Res, 2000
- Nerve growth factor, brain derived neurotrophic factor  [ **BDNF** ]   **BDNF**  and neurotrophin 3  [ **NTF3** ]  NT 3  [ **NTF3** ]  mRNAs were revealed in OLGs in vivo and in culture.  Ref: 12843289 J Neurosci, 2003
